# Analysis of the Serum Bile Acid Composition for Differential Diagnosis in Patients with Liver Disease

**DOI:** 10.1155/2015/717431

**Published:** 2015-03-03

**Authors:** Tomonori Sugita, Katsushi Amano, Masanori Nakano, Noriko Masubuchi, Masahiro Sugihara, Tomokazu Matsuura

**Affiliations:** ^1^Division of Gastroenterology and Hepatology, Department of Internal Medicine, The Jikei University School of Medicine, 3-25-8 Nishi-shinbashi, Minato, Tokyo 105-8461, Japan; ^2^Drug Metabolism & Pharmacokinetics Research Laboratories, R&D Division, Daiichi Sankyo Co., Ltd., Tokyo, Japan; ^3^Clinical Data & Biostatistics Department, R&D Division, Daiichi Sankyo Co., Ltd., Tokyo, Japan; ^4^Department of Laboratory Medicine, The Jikei University School of Medicine, Tokyo, Japan

## Abstract

*Objectives*. We determined the serum bile acid (BA) composition in patients with liver diseases and healthy volunteers to investigate the relationship between the etiologies of liver disease and BA metabolism.* Material and Methods*. Sera from 150 patients with liver diseases and 46 healthy volunteers were obtained. The serum concentrations of the 16 different BAs were determined according to the LC-MS/MS method and were compared between the different liver diseases.* Results*. A total of 150 subjects, including patients with hepatitis C virus (HCV) (*n* = 44), hepatitis B virus (HBV) (*n* = 23), alcoholic liver disease (ALD) (*n* = 21), biliary tract disease (*n* = 20), nonalcoholic fatty liver disease (NAFLD) (*n* = 13), and other liver diseases (*n* = 29), were recruited. The levels of UDCA and GUDCA were significantly higher in the ALD group, and the levels of DCA and UDCA were significantly lower in the biliary tract diseases group than in viral hepatitis group. In the UDCA therapy (−) subgroup, a significantly lower level of TLCA was observed in the ALD group, with lower levels of CDCA, DCA, and GLCA noted in biliary tract diseases group compared to viral hepatitis group.* Conclusions*. Analysis of the BA composition may be useful for differential diagnosis in liver disease.

## 1. Introduction

Many blood biochemical markers, such as AST, ALT, gamma GTP, and ALP, are utilized to evaluate the liver function. Most of these classical blood biochemical markers are enzymes and are elevated as a result of hepatic cell injury. Thus, these tests cannot be expected to provide a definitive diagnosis of liver disease. The development of more specific liver function tests is desired to differentiate one form of hepatitis from another, or to determine whether the cholestasis is intra- or extrahepatic.

Bile acids (BAs) are the largest organic components in bile and are synthesized from cholesterol in the liver. BAs play an important role in the elimination of cholesterol from the body and are also crucial for lipid absorption. Two primary BAs, cholic acid (CA) and chenodeoxycholic acid (CDCA), are synthesized from cholesterol in the liver [1]. After being secreted into the small intestine through the bile duct, deoxycholic acid (DCA), lithocholic acid (LCA), and ursodeoxycholic acid (UDCA), which are considered to be secondary BAs, are converted from primary BAs by the action of intestinal flora [2]. Free BAs are easily conjugated with glycine or taurine in the liver [3]. About 95% of primary and secondary BAs are reabsorbed from the ileum, returning to the liver by portal circulation and then are secreted into bile again via a process called enterohepatic circulation [2].

Some BAs leak into the systemic circulation, but under normal physiological conditions, the serum BA concentrations are much lower than those of the bile due to efficient first-pass extraction [4]. On the other hand, in liver and intestinal diseases, the serum BA concentrations are changed due to the impairment of hepatic synthesis and extraction of BAs or to changes in the intestinal absorption. Since liver diseases can affect BA synthesis and metabolism, the serum BA concentration has been utilized as a prognostic and diagnostic marker for some diseases, such as recurrent intrahepatic cholestatic jaundice of pregnancy [5–7]. However, the detailed metabolism of BAs in patients with liver diseases, especially those associated with the different etiologies of liver diseases, remains to be clarified.

In this study, we determined the serum BA composition in patients with several liver diseases and healthy volunteers according to a LC-MS/MS method in order to investigate the relationship between the etiologies of liver diseases and BA metabolism.

## 2. Materials and Methods

### 2.1. Patients and Healthy Controls

A total of 150 patients with liver diseases who visited The Jikei University Hospital from March 2011 to March 2013 and 46 healthy volunteers were recruited. The background of healthy volunteers was as follows. They consisted of 25 males and 21 females, the age ranged from 20 to 39 years old, the BMI was within the Japanese normal range (18.5~25.0 kg/m^2^), and the average amount of ethanol was less than 20 g per day. None of them had diabetes and dyslipidemia in the most recent medical examination. They were not diagnosed as gallstone and any liver dysfunction and never had a medication of UDCA in the past.

This clinical study was carried out with the approval of the Ethics Committee of The Jikei University School of Medicine, and written informed consent was obtained from all patients and healthy volunteers.

### 2.2. Sampling and Measurement of the Serum BA Levels

The sera of patients and healthy volunteers were obtained using their fasting blood in the early morning. The serum concentrations of the following 16 BAs were determined by a LC-MS/MS method: cholic acid (CA), chenodeoxycholic acid (CDCA), deoxycholic acid (DCA), glycocholic acid (GCA), glycochenodeoxycholic acid (GCDCA), glycodeoxycholic acid (GDCA), glycolithocholic acid (GLCA), glycoursodeoxycholic acid (GUDCA), lithocholic acid (LCA), taurocholic acid (TCA), taurochenodeoxycholic acid (TCDCA), taurodeoxycholic acid (TDCA), taurolithocholic acids (TLCA), tauroursodeoxycholic acid (TUDCA), ursodeoxycholic acid (UDCA), and 12-ketolithocholic acid (12-KLCA). The LC system was an ACQUITY Ultra Performance LC (Waters). The LC was connected to a Xevo TQ MS (Waters). HPLC was performed on an ACQUITY UPLC BEH C18, 1.7 *μ*m, 2.1 × 50 mm column (Waters), and the column temperature was maintained at 50°C.

Individual BAs were eluted with a gradient at a flow rate of 0.8 mL/min. Mobile phase A was water/formic acid (1000 : 1, v/v) and mobile phase B was acetonitrile. The samples were eluted with 80% mobile phase A and 20% mobile phase B for an initial 0.30 min after injection, then with a linear gradient of mobile phase B of 20% to 30% over 5.00 min, followed by mobile phase B at 80% over 8.50 min, which was held for 0.50 min. Before the injection of the next sample, the column was equilibrated with 80% mobile phase A for 1 min. After centrifugation of the mixture with 20 uL of serum sample, 80 *μ*L of ethanol and 20 *μ*L of IS (naptalam) solution, 10 *μ*L of the supernatant with water/formic acid (1000 : 1, v/v) solution was injected into the LC/MS/MS system. The method was validated ranging from 0.010–30 nmol/mL. The mass spectrometer was equipped with an electrospray source operated in the negative ion mode using the selected ion monitoring mode.

We also examined the following biochemical markers in the serum samples: AST, ALT, ALP, *γ*-GTP, albumin (ALB), total bilirubin (T-Bil), direct bilirubin (D-Bil), prothrombin time (PT), and total bile acids (TBA). Total bile acid was measured independently by enzyme cycling method.

### 2.3. Statistical Analysis

Descriptive statistics were used to summarize the patient characteristics. The concentrations for the biochemical data and the BAs were log-transformed to approximately normalize the distributions. The least square geometric mean of the biochemical data and the BAs were estimated for the different etiologies of liver diseases and were compared between them using multiple linear regression models adjusted for sex, age, body mass index (BMI), alcohol consumption, type of liver disease, dyslipidemia, diabetes mellitus, and the use of UDCA. A significance level of 0.05 was used for all statistical tests, and two-tailed tests were applied. All statistical analyses were performed using the SAS software program, version 9.2 (SAS Institute, Cary, NC).

## 3. Results

### 3.1. Clinical Patient Characteristics

A total of 150 subjects, including patients with hepatitis C virus infection (*n* = 44), hepatitis B virus infection (*n* = 23), alcoholic liver disease (ALD) (*n* = 21), biliary tract disease (*n* = 20), nonalcoholic fatty liver disease (NAFLD) (*n* = 13), autoimmune hepatitis (AIH) (*n* = 6), primary biliary cirrhosis (PBC) (*n* = 8), liver abscess (*n* = 3), drug-induced liver injury (*n* = 2), cytomegalovirus (CMV) hepatitis (*n* = 2), and unknown etiology (*n* = 8) were recruited. The 20 patients with biliary tract disease included those with biliary tract stones (*n* = 11), obstructive jaundice due to malignancy (*n* = 8), and primary sclerosing cholangitis (PSC) (*n* = 1). The patient characteristics classified by the etiologies of the liver diseases are shown in Table 1. Acute hepatitis/liver damage type was observed more frequently in patients with biliary tract disease than in those with other diseases. The percentage of UDCA therapy was higher in patients with viral hepatitis than in those with other diseases. More than half of the patients with NAFLD had dyslipidemia or DM.

### 3.2. Biochemical Data and BA Composition

The results of the multiple linear regression analyses for the biochemical data classified by liver diseases are shown in Table 2. Significantly higher levels of serum ALP, gamma GTP, and bilirubin were observed in the patients with biliary tract diseases than in those with viral hepatitis (ALP: 566.0 U/L versus 279.4 U/L (*P* < 0.01); gamma GTP: 224.1 U/L versus 70.4 U/L (*P* < 0.01); T-Bil: 2.09 mg/dL versus 1.18 mg/dL (*P* < 0.05); D-Bil: 0.49 mg/dL versus 0.15 mg/dL (*P* < 0.05)). In contrast, the serum Alb level was significantly lower in patients with biliary tract diseases than in those with viral hepatitis (3.09 g/dL versus 3.65 g/dL (*P* < 0.01)).

The results of the multiple linear regression analyses of the serum BA composition of patients classified by liver diseases are shown in Table 3. The levels of UDCA and GUDCA were significantly higher in the patients with ALD than in those with viral hepatitis (UDCA: 1.15 *μ*M versus 0.237 *μ*M, (*P* < 0.01); GUDCA: 3.34 *μ*M versus 0.900 *μ*M (*P* = 0.02)). On the other hand, the DCA and UDCA levels were significantly lower in the patients with biliary tract disease than in those with viral hepatitis (DCA: 0.032 *μ*M versus 0.129 *μ*M, (*P* < 0.01); UDCA: 0.088 *μ*M versus 0.237 *μ*M (*P* = 0.03)). The TCA level was significantly higher in the patients with biliary tract disease than in those with viral hepatitis (TCA: 0.690 *μ*M versus 0.229 *μ*M, (*P* < 0.05)). Since the number of patients with biliary tract disease, alcoholic liver disease, NAFLD, and other liver diseases was too small, there was no significant difference between groups.

A subgroup analysis was performed to eliminate the effect of UDCA therapy, and the results are shown in Tables 4(a) and 4(b). In the UDCA therapy (−) group, a significantly lower level of TLCA in the ALD patients, and CDCA, DCA, and GLCA levels in the patients with biliary tract diseases were observed compared to the levels in patients with viral hepatitis (TLCA: 0.0034 *μ*M versus 0.0183 *μ*M (*P* < 0.01); CDCA: 0.0532 *μ*M versus 0.187 *μ*M (*P* < 0.05); DCA: 0.0284 versus 0.126 *μ*M (*P* < 0.05); GLCA: 0.0098 *μ*M versus 0.0234 *μ*M (*P* < 0.01)). The UDCA level in patients with ALD was significantly higher than that in patients with viral hepatitis (0.335 *μ*M versus 0.0505 *μ*M (*P* < 0.01)). To present comparison between healthy control and liver disease group, the percentage of patients whose bile acid concentration and biochemical data exceed 97.5% cut-off value of healthy control is shown in Tables 5(a) and 5(b).

Contrasting results were observed in the UDCA therapy (+) group. Significantly higher levels of GCA and GCDCA were observed in patients with ALD, biliary tract diseases, and NAFLD compared to those with viral hepatitis (GCA: 2.57 *μ*M versus 3.44 *μ*M versus 3.86 *μ*M versus 0.556 *μ*M (*P* < 0.05); GCDCA: 8.14 *μ*M versus 10.0 *μ*M versus 13.0 *μ*M versus 2.46 *μ*M (*P* < 0.05)). A higher level of TCA was also observed in the patients with ALD and biliary tract diseases compared to those with viral hepatitis (0.757 *μ*M versus 1.07 *μ*M versus 0.148 *μ*M (*P* < 0.05)). The serum level of several other BAs, such as CA in the NAFLD patients, TCDCA in the patients with biliary tract diseases, and GUDCA and TUDCA in the ALD patients, was higher than those in patients with viral hepatitis (CA: 1.27 *μ*M versus 0.0788 *μ*M (*P* < 0.05); TCDCA: 4.18 *μ*M versus 0.681 *μ*M (*P* < 0.05); GUDCA: 21.8 *μ*M versus 4.24 *μ*M (*P* < 0.05); TUDCA: 2.49 *μ*M versus 0.454 *μ*M (*P* < 0.05)).

## 4. Discussion

Since BA synthesis and metabolism are affected by liver diseases, the BAs and their composition have been studied and utilized as diagnostic and prognostic markers. However, it has been unclear how the etiologies of liver diseases affect the BA composition. In this study, we investigated the serum BA compositions, including the levels of conjugated BAs, using LC-MS/MS in a large number of patients with different etiologies of liver diseases. In healthy human controls, the serum BA composition, including the conjugated BAs, determined according to the LC-MS/MS method has recently been reported [4, 8, 9]. Bathena et al. reported that the BAs were dominated by CDCA and DCA in the serum, and amidation with glycine was predominant over taurine. They reported that 55% of the serum BAs were conjugated with glycine, and 13% of the serum BAs were conjugated with taurine [8]. In our study, GCDCA was the predominant BA of the major BAs (CA, CDCA, DCA, LCA and UDCA, and the amidation products of these BAs with glycine or taurine) in healthy controls as well and was also predominant in patients with liver disease of all etiologies in the UDCA therapy (−) group.

In healthy controls, the serum concentrations of BAs were much lower than those of patients with liver diseases, because BAs are rarely noted in the systemic circulation due to enterohepatic circulation with efficient first-pass extraction. In the case of some liver diseases, such as PBC and obstructive jaundice, which cause disorders of BA excretion, the serum BA concentrations were markedly increased [10]. The types of liver disease (acute or chronic), especially liver cirrhosis, also affect the BA metabolism. In cirrhotic patients, it has been reported that the serum BA concentrations are increased due to the impairment of bile production and secretion [10].

**Figure 1:**
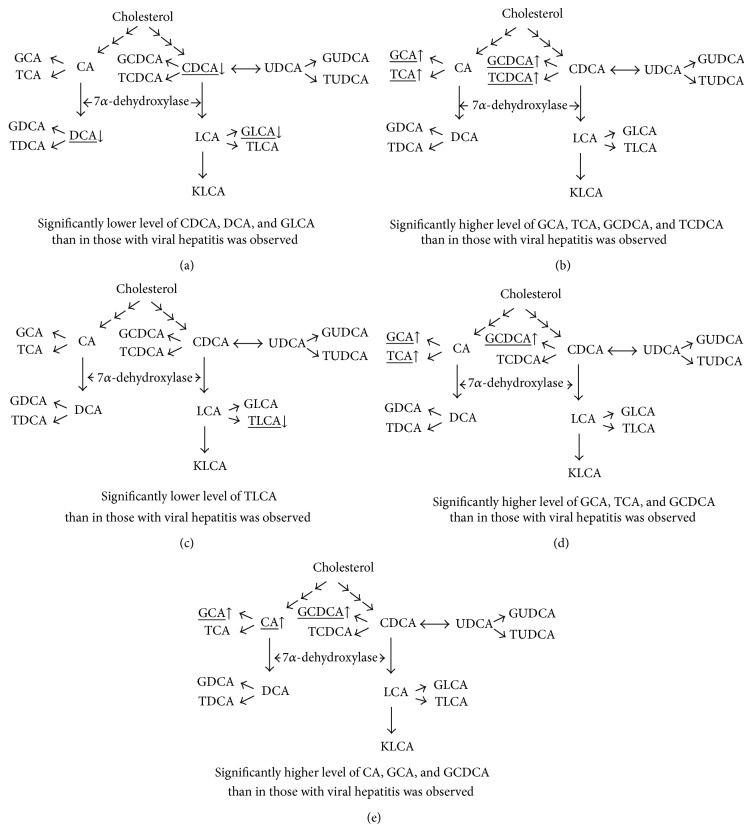
(a) Bile acid composition in patients with biliary tract disease (without UDCA treatment). (b) Bile acid composition in patients with biliary tract disease (with UDCA treatment). (c) Bile acid composition in patients with ALD (without UDCA treatment). (d) Bile acid composition in patients with ALD (with UDCA treatment). (e) Bile acid composition in patients with NAFLD (with UDCA treatment).

Particularly in patients with cholestatic liver diseases, the serum BAs levels are utilized as biomarkers [5]. Trottier et al. reported that the levels of taurine and glycine conjugates of primary BAs were elevated in both patients with PBC and PSC compared to noncholestatic donors [11]. In our study, the levels of BAs conjugated with taurine and glycine were elevated in patients with all kinds of liver diseases compared to healthy volunteers. Thus, the elevation of the levels of taurine- and glycine-conjugated BAs may not be disease specific. On the other hand, there were significantly lower levels of CDCA, DCA, and GLCA in patients with biliary tract disease without UDCA treatment than in those with viral hepatitis in our study (Figure 1(a)). Trottier et al. also reported that the levels of secondary BAs, such as DCA and LCA, were reduced in patients with PSC when compared with noncholestatic patients and patients with PBC [11]. These results may imply that there is a significant impairment of dehydroxylation to secondary BA in patients with biliary tract diseases, since secondary BAs are formed through an enzymatic dehydroxylation of primary BAs catalyzed by bacterial enzymes in the intestine.

It is reasonable that the impairment of BA excretion from the liver into the intestine, especially that due to extrahepatic obstruction, results in decreased level of secondary BAs. In contrast, the glycine- and taurine-conjugated forms of primary BAs were significantly elevated in patients with biliary tract disease receiving UDCA treatment compared to patients with viral hepatitis (Figure 1(b)). In patients with biliary tract disease, enhancement of BA excretion by UDCA may lead to a significant elevation of primary BAs in the blood circulation because of the impairment of dehydroxylation to secondary BAs. In our study, the TLCA levels in patients with ALD were significantly decreased compared to those in patients with viral hepatitis (Figure 1(c)). Although the differences were not statistically significant, all of the other forms of taurine-conjugated BAs were also decreased in patients with ALD. The altered expression of genes related to BA metabolism and the changes in the gut microbiota due to ethanol consumption may be possible explanations for our results.

In support of this, Xie et al. reported that ethanol consumption led to a significant elevation of unconjugated and glycine-conjugated BAs and a reduction of taurine-conjugated BAs in rats. In their report, ethanol consumption altered the expression of genes related to BA metabolism and BA transport in the liver and ileum in rats. They also mentioned the possibility that the reduced taurine-conjugated BAs in ethanol-treated rats were partially due to an ethanol-induced disturbance of the gut microbiota [12]. In addition, it has been reported that ethanol consumption promotes the overgrowth of bacteria in the small intestine [13, 14].

Recently, BAs have been discovered to play an important role in the regulation of the metabolism of glucose and lipids in humans [15–19] through the activation of the farnesoid X receptor (FXR) [20, 21], which is a nuclear receptor, and TGR5 [22–24], which is a membrane receptor. Lake et al. reported that there were elevated levels of taurine-conjugated BAs and decreased levels of CA and GDCA in the livers from patients with NASH. They also revealed that there was a potential shift toward the alternative pathway of BA synthesis during NASH based on a transcriptomic analysis of 70 BAs. They postulated that the transcriptomic changes in the BA synthesis pathway enzymes, together with altered hepatic BA composition, signify an attempt by the liver to reduce the hepatotoxicity during disease progression to NASH [25].

In our study, the glycine- or taurine-conjugated primary BAs levels were more elevated in patients with ALD and NAFLD than in those with viral hepatitis on UDCA treatment. For example, GCA, TCA, and GCDCA were significantly elevated in patients with ALD on UDCA treatment (Figure 1(d)). In addition, the levels of CA, GCA, and GCDCA were significantly elevated in patients with NAFLD on UDCA treatment (Figure 1(e)). The exact mechanisms underlying the changes in BA composition induced by UDCA treatment are unclear, but dyslipidemia and diabetes were observed less frequently in patients with viral hepatitis in our study and that may have had an influence on the differences in the BA composition between the patients with viral hepatitis and other liver diseases.

In conclusion, our study showed that the BA composition differed between the patients with different liver disease etiologies. Analyses of the BA composition may be useful for differential diagnosis of liver diseases.

## Figures and Tables

**Table 1:** The patient characteristics.

	Viral hepatitis (HBV + HCV)	ALD	Biliary tract disease	NAFLD	Other liver diseases	Total
	(*n* = 67)	(*n* = 21)	(*n* = 20)	(*n* = 13)	(*n* = 29)	(*n* = 150)
Sex (male)	40 (59.7%)	20 (95.2%)	14 (70.0%)	6 (46.2%)	9 (31.0%)	89 (59.3%)
Age (Yr)^*^	59.9 ± 16.3	61.0 ± 12.7	66.5 ± 11.6	62.5 ± 16.5	58.0 ± 15.2	60.8 ± 15.1
BMI (Kg/m^2^)^*^	23.4 ± 4.0	23.5 ± 4.3	23.6 ± 3.3	25.5 ± 2.8	22.2 ± 2.7	23.4 ± 3.7
Alcohol consumption (>20 g/day)	8 (11.9%)	17 (81.0%)	4 (20.0%)	0 (0.0%)	5 (17.2%)	34 (22.7%)

Type of liver disease (% in each etiology)						
Acute hepatitis/liver damage	6 (9.0%)	1 (4.7%)	11 (55.0%)	0 (0.0%)	11 (37.9%)	29 (19.3%)
Chronic hepatitis/liver damage	32 (47.7%)	9 (42.9%)	4 (20.0%)	9 (69.2%)	14 (48.3%)	68 (45.3%)
Liver cirrhosis	11 (16.4%)	5 (23.8%)	0 (0.0%)	1 (7.7%)	2 (6.9%)	19 (12.7%)
With HCC	18 (26.9%)	6 (28.6%)	5 (25.0%)	3 (23.1%)	2 (6.9%)	34 (22.7%)

Complications (% in each etiology)						
Obstructive jaundice	1 (1.5%)	0 (0.0%)	6 (30.0%)	0 (0.0%)	0 (0.0%)	7 (4.7%)
Biliary tract stone	8 (11.9%)	6 (28.6%)	13 (65.0%)	1 (7.7%)	3 (10.3%)	31 (20.7%)
Dyslipidemia	7 (10.4%)	5 (23.8%)	8 (40.0%)	9 (69.2%)	4 (13.8%)	33 (22.0%)
DM	8 (11.9%)	9 (42.9%)	5 (25.0%)	7 (53.8%)	3 (10.3%)	31 (20.7%)

UDCA therapy	40 (59.7%)	9 (42.9%)	5 (25.0%)	3 (23.1%)	11 (38.0%)	68 (45.3%)

^*^The values are the means ± standard deviation or numbers (%).

**Table 2:** The biochemical data for the patients with different liver diseases.

	Viral hepatitis (HBV + HCV)	ALD	Biliary tract disease	NAFLD	Other liver diseases	Healthy controls
ALT (U/L)	77.1 (59.0, 100.8)	79.7 (49.6, 128.0)	79.7 (49.6, 128.0)	64.1 (34.2, 120.0)	57.4 (38.5, 85.6)	12.0 (6.0, 23.0)
AST (U/L)	74.9 (59.3, 94.5)	54.9 (34.4, 87.5)	67.3 (44.5, 101.7)	46.2 (26.8, 79.8)	62.0 (43.7, 87.8)	19.0 (14.0, 32.0)
ALP (U/L)	279.4 (241.8, 322.9)	347.4 (260.1, 464.0)	566.0 (438.0, 731.3)^**^	261.8 (186.5, 367.5)	433.5 (349.3, 538.0)^**^	163.5 (121.0, 291.0)
Γ-GTP (U/L)	70.4 (55.4, 89.6)	113.6 (70.2, 183.8)	224.1 (146.3, 343.2)^**^	45.1 (25.7, 79.3)	102.9 (71.9, 147.4)	16.0 (11.0, 32.0)
T-Bil (mg/dL)	1.18 (0.95, 1.46)	1.05 (0.69, 1.62)	2.09 (1.43, 3.05)^*^	1.19 (0.72, 1.97)	1.41 (1.02, 1.94)	0.8 (0.5, 1.9)
D-Bil (mg/dL)	0.15 (0.10, 0.23)	0.12 (0.05, 0.29)	0.49 (0.22, 1.06)^*^	0.12 (0.04, 0.33)	0.16 (0.08, 0.31)	0.1 (0.0, 0.1)
TBA (*μ*M)	14.57 (10.55, 20.11)	15.67 (8.27, 29.70)	22.64 (12.64, 40.56)	13.04 (6.03, 28.20)	15.60 (9.59, 25.37)	2.8 (1.0, 15.1)
ALB (g/dL)	3.65 (3.49, 3.82)	3.45 (3.16, 3.77)	3.09 (2.86, 3.35)^**^	3.77 (3.40, 4.18)	3.25 (3.03, 3.47)^**^	4.8 (4.3, 5.2)
PT (%)	80.9 (76.4, 85.7)	84.2 (75.0, 94.4)	82.3 (74.2, 91.3)	86.5 (75.6, 99.0)	76.3 (70.1, 83.1)	93.0 (71.0, 112.0)

The values in patients with liver disease are the least square geometric mean concentrations with 95% CI.

The values in healthy controls are the median with 2.5% point and 97.5% point.

^**^ *P* < 0.01 and ^*^ *P* < 0.05 versus viral hepatitis in patients with liver disease.

**Table 3:** The serum bile acid compositions among the patients with different liver diseases.

(*μ*M)	Viral hepatitis (HBV + HCV)	ALD	Biliary tract disease	NAFLD	Other liver diseases	Healthy controls
*n*	67	21	20	13	29	46

CA	0.0851 (0.0560, 0.1293)	0.1303 (0.0564, 0.3008)	0.0695 (0.0331, 0.1459)	0.1867 (0.0700, 0.4978)	0.0821 (0.0439, 0.1533)	0.0638 (0.0129, 0.9090)
GCA	0.8171 (0.5323, 1.2543)	0.9344 (0.3964, 2.2028)	1.9652 (0.9196, 4.2000)	0.6518 (0.2387, 1.7800)	1.4384 (0.7584, 2.7280)	0.1235 (0.0206, 2.7400)
TCA	0.2289 (0.1368, 0.3828)	0.1952 (0.0697, 0.5462)	0.6900 (0.2774, 1.7164)^*^	0.1147 (0.0344, 0.3830)	0.4915 (0.2280, 1.0594)	0.0157 (0.0016, 0.9240)
CDCA	0.2539 (0.1640, 0.3931)	0.5525 (0.2304, 1.3251)	0.1047 (0.0483, 0.2272)	0.5511 (0.1978, 1.5357)	0.1578 (0.0821, 0.3032)	0.3445 (0.0294, 2.3400)
GCDCA	2.8356 (2.0016, 4.0171)	3.7629 (1.8744, 7.5538)	4.9012 (2.6441, 9.0851)	2.6327 (1.1638, 5.9558)	3.3493 (1.9911, 5.6341)	0.5460 (0.1130, 4.3400)
TCDCA	0.8308 (0.5354, 1.2891)	0.7649 (0.3176, 1.8421)	1.9965 (0.9167, 4.3483)	0.3995 (0.1427, 1.1186)	1.1573 (0.6006, 2.2301)	0.1095 (0.0177, 1.1900)
DCA	0.1290 (0.0791, 0.2105)	0.0788 (0.0296, 0.2096)	0.0322 (0.0135, 0.0766)^**^	0.1984 (0.0630, 0.6245)	0.0736 (0.0355, 0.1528)	0.2665 (0.0000, 0.7360)
GDCA	0.2320 (0.1334, 0.4033)	0.1449 (0.0479, 0.4382)	0.1782 (0.0669, 0.4748)	0.2530 (0.0692, 0.9250)	0.1786 (0.0782, 0.4080)	0.1570 (0.0000, 1.8200)
TDCA	0.0767 (0.0499, 0.1181)	0.0390 (0.0165, 0.0924)	0.0900 (0.0419, 0.1933)	0.0627 (0.0228, 0.1722)	0.0635 (0.0334, 0.1209)	0.0219 (0.0000, 0.3980)
LCA	0.0242 (0.0184, 0.0318)	0.0220 (0.0127, 0.0381)	0.0177 (0.0109, 0.0289)	0.0346 (0.0182, 0.0659)	0.0178 (0.0118, 0.0269)	0.0103 (0.0000, 0.0291)
GLCA	0.0233 (0.0173, 0.0313)	0.0142 (0.0079, 0.0256)	0.0150 (0.0089, 0.0253)	0.0276 (0.0138, 0.0550)	0.0229 (0.0147, 0.0355)	0.0071 (0.0000, 0.0338)
TLCA	0.0145 (0.0105, 0.0200)	0.0109 (0.0057, 0.0207)	0.0176 (0.0100, 0.0311)	0.0095 (0.0045, 0.0201)	0.0140 (0.0087, 0.0226)	0.0057 (0.0000, 0.0231)
UDCA	0.2369 (0.1539, 0.3645)	1.1532 (0.4868, 2.7321)^**^	0.0884 (0.0412, 0.1897)^*^	0.3263 (0.1188, 0.8962)	0.2254 (0.1184, 0.4290)	0.1975 (0.0000, 0.6030)
GUDCA	0.8998 (0.5728, 1.4134)	3.3367 (1.3518, 8.2361)^*^	0.7939 (0.3567, 1.7672)	0.7276 (0.2525, 2.0970)	1.0877 (0.5542, 2.1349)	0.2110 (0.0145, 1.4200)
TUDCA	0.1076 (0.0659, 0.1756)	0.2419 (0.0908, 0.6450)	0.1281 (0.0537, 0.3052)	0.0553 (0.0175, 0.1745)	0.1516 (0.0729, 0.3151)	0.0085 (0.0000, 0.1510)
12-KLCA	0.0561 (0.0436, 0.0723)	0.0397 (0.0239, 0.0657)	0.0355 (0.0227, 0.0555)	0.0604 (0.0334, 0.1092)	0.0322 (0.0221, 0.0469)	0.0101 (0.0000, 0.0943)

Cumulative total bile acid	6.8515	11.4215	11.1237	6.2919	8.5437	2.0986

The values in patients with liver disease are the least square geometric mean concentrations with 95% CI.

The values in healthy controls are the median with 2.5% point and 97.5% point.

^**^ *P* < 0.01 and ^*^ *P* < 0.05 versus viral hepatitis in patients with liver disease.

**(a):** 

(*μ*M)	Viral hepatitis (HBV + HCV)	ALD	Biliary tract disease	NAFLD	Other liver diseases	Healthy controls
*n*	40	9	5	3	11	46

CA	0.0788 (0.0440, 0.1413)	0.1851 (0.0462, 0.7409)	0.1414 (0.0276, 0.7236)	1.2708 (0.1102, 14.649)^*^	0.2271 (0.0745, 0.6921)	0.0638 (0.0129, 0.9090)
GCA	0.5557 (0.3718, 0.8306)	2.5674 (0.9881, 6.6709)^**^	3.4416 (1.1186, 10.589)^**^	3.8566 (0.7168, 20.750)^*^	1.0244 (0.4757, 2.2061)	0.1235 (0.0206, 2.7400)
TCA	0.1479 (0.0873, 0.2503)	**0.7570 (0.2167, 2.6445)** ^*^	1.0705 (0.2456, 4.6665)^*^	0.7062 (0.0779, 6.4018)	0.2248 (0.0823, 0.6141)	0.0157 (0.0016, 0.9240)
CDCA	0.3485 (0.1975, 0.6151)	0.5274 (0.1368, 2.0341)	0.3220 (0.0658, 1.5771)	3.7919 (0.3514, 40.922)	0.7003 (0.2368, 2.0715)	0.3445 (0.0294, 2.3400)
GCDCA	2.4618 (1.7090, 3.5462)	8.1448 (3.4220, 19.386)^*^	10.005 (3.6055, 27.763)^*^	13.048 (2.8304, 60.149)^*^	3.6051 (1.7962, 7.2358)	0.5460 (0.1130, 4.3400)
TCDCA	0.6805 (0.4143, 1.1177)	2.4085 (0.7408, 7.8300)	4.1830 (1.0444, 16.754)^*^	2.0567 (0.2575, 16.426)	0.7495 (0.2907, 1.9326)	0.1095 (0.0177, 1.1900)
DCA	0.1406 (0.0736, 0.2686)	0.0804 (0.0173, 0.3742)	0.0356 (0.0058, 0.2174)	0.0830 (0.0055, 1.2468)	0.1628 (0.0473, 0.5600)	0.2665 (0.0000, 0.7360)
GDCA	0.2278 (0.1124, 0.4618)	0.1826 (0.0341, 0.9781)	0.0482 (0.0067, 0.3474)	0.1359 (0.0071, 2.6181)	0.2567 (0.0666, 0.9888)	0.1570 (0.0000, 1.8200)
TDCA	0.0624 (0.0355, 0.1099)	0.0620 (0.0162, 0.2376)	0.0509 (0.0105, 0.2472)	0.0599 (0.0056, 0.6386)	0.0411 (0.0140, 0.1209)	0.0219 (0.0000, 0.3980)
LCA	0.0275 (0.0186, 0.0408)	0.0283 (0.0111, 0.0719)	0.0165 (0.0055, 0.0494)	0.0520 (0.0101, 0.2688)	0.0392 (0.0185, 0.0829)	0.0103 (0.0000, 0.0291)
GLCA	0.0252 (0.0159, 0.0401)	0.0219 (0.0073, 0.0657)	0.0293 (0.0080, 0.1069)	0.0305 (0.0044, 0.2114)	0.0299 (0.0124, 0.0723)	0.0071 (0.0000, 0.0338)
TLCA	0.0149 (0.0098, 0.0228)	0.0198 (0.0073, 0.0541)	0.0213 (0.0065, 0.0694)	0.0078 (0.0013, 0.0455)	0.0099 (0.0044, 0.0221)	0.0057 (0.0000, 0.0231)
UDCA	1.5203 (0.8882, 2.6022)	5.1120 (1.4256, 18.330)	0.8803 (0.1958, 3.9572)	1.2878 (0.1357, 12.224)	2.6510 (0.9503, 7.3956)	0.1975 (0.0000, 0.6030)
GUDCA	4.2367 (2.6009, 6.9014)	21.824 (6.8462, 69.568)^*^	9.8971 (2.5289, 38.734)	5.9534 (0.7717, 45.926)	6.1963 (2.4413, 15.727)	0.2110 (0.0145, 1.4200)
TUDCA	0.4544 (0.2526, 0.8173)	2.4936 (0.6179, 10.063)^*^	1.4632 (0.2833, 7.5587)	0.2802 (0.0240, 3.2756)	0.4095 (0.1335, 1.2561)	0.0085 (0.0000, 0.1510)
12-KLCA	0.0707 (0.0469, 0.1067)	0.0319 (0.0120, 0.0846)	0.0796 (0.0252, 0.2511)	0.1449 (0.0259, 0.8101)	0.0469 (0.0214, 0.1029)	0.0101 (0.0000, 0.0943)

The values in patients with liver disease are the least square geometric mean concentrations with 95% CI.

The values in healthy controls are the median with 2.5% point and 97.5% point.

^**^ *P* < 0.01 and ^*^ *P* < 0.05 versus viral hepatitis in patients with liver disease.

**(b):** 

(*μ*M)	Viral hepatitis (HBV + HCV)	ALD	Biliary tract disease	NAFLD	Other liver diseases	Healthy controls
*n*	27	12	15	10	18	46

CA	0.1038 (0.0536, 0.2008)	0.1134 (0.0328, 0.3918)	0.0461 (0.0201, 0.1055)	0.1123 (0.0384, 0.3284)	0.0398 (0.0187, 0.0844)	0.0638 (0.0129, 0.9090)
GCA	1.2768 (0.5390, 3.0246)	0.4700 (0.0930, 2.3740)	1.6582 (0.5622, 4.8914)	0.5272 (0.1297, 2.1427)	1.6791 (0.6277, 4.4916)	0.1235 (0.0206, 2.7400)
TCA	0.4047 (0.1493, 1.0971)	0.0527 (0.0081, 0.3428)	0.6966 (0.1994, 2.4335)	0.1199 (0.0237, 0.6067)	0.6846 (0.2194, 2.1360)	0.0157 (0.0016, 0.9240)
CDCA	0.1874 (0.0898, 0.3912)	0.8048 (0.2020, 3.2062)	0.0532 (0.0211, 0.1339)^*^	0.2408 (0.0728, 0.7968)	0.0583 (0.0252, 0.1350)^*^	0.3445 (0.0294, 2.3400)
GCDCA	3.3958 (1.7277, 6.6742)	2.6308 (0.7395, 9.3586)	3.4186 (1.4648, 7.9788)	1.7610 (0.5870, 5.2829)	3.0607 (1.4158, 6.6167)	0.5460 (0.1130, 4.3400)
TCDCA	1.1414 (0.5019, 2.5960)	0.2730 (0.0583, 1.2776)	1.6008 (0.5711, 4.4867)	0.3453 (0.0908, 1.3133)	1.3209 (0.5173, 3.3731)	0.1095 (0.0177, 1.1900)
DCA	0.1264 (0.0552, 0.2892)	0.0840 (0.0177, 0.3980)	0.0284 (0.0100, 0.0801)^*^	0.2234 (0.0581, 0.8586)	0.0428 (0.0167, 0.1102)	0.2665 (0.0000, 0.7360)
GDCA	0.2337 (0.0891, 0.6126)	0.1327 (0.0217, 0.8109)	0.2669 (0.0797, 0.8940)	0.3061 (0.0639, 1.4669)	0.1427 (0.0475, 0.4284)	0.1570 (0.0000, 1.8200)
TDCA	0.0984 (0.0466, 0.2076)	0.0244 (0.0060, 0.0992)	0.1251 (0.0490, 0.3192)	0.0758 (0.0225, 0.2552)	0.0791 (0.0337, 0.1854)	0.0219 (0.0000, 0.3980)
LCA	0.0238 (0.0158, 0.0357)	0.0151 (0.0070, 0.0323)	0.0175 (0.0105, 0.0291)	0.0328 (0.0169, 0.0635)	0.0095 (0.0060, 0.0151)^**^	0.0103 (0.0000, 0.0291)
GLCA	0.0234 (0.0160, 0.0342)	0.0114 (0.0056, 0.0233)	0.0098 (0.0061, 0.0158)^**^	0.0266 (0.0143, 0.0493)	0.0179 (0.0116, 0.0276)	0.0071 (0.0000, 0.0338)
TLCA	0.0183 (0.0107, 0.0314)	0.0034 (0.0012, 0.0094)^**^	0.0178 (0.0091, 0.0348)	0.0138 (0.0058, 0.0330)	0.0145 (0.0079, 0.0267)	0.0057 (0.0000, 0.0231)
UDCA	0.0505 (0.0233, 0.1097)	0.3348 (0.0781, 1.4358)^*^	0.0178 (0.0067, 0.0470)	0.0800 (0.0227, 0.2822)	0.0308 (0.0127, 0.0747)	0.1975 (0.0000, 0.6030)
GUDCA	0.2576 (0.1052, 0.6310)	0.8175 (0.1520, 4.3961)	0.1544 (0.0502, 0.4750)	0.1648 (0.0384, 0.7069)	0.2442 (0.0879, 0.6787)	0.2110 (0.0145, 1.4200)
TUDCA	0.0383 (0.0157, 0.0936)	0.0251 (0.0047, 0.1341)	0.0217 (0.0051, 0.0926)	0.0217 (0.0051, 0.0926)	0.0463 (0.0167, 0.1281)	0.0085 (0.0000, 0.1510)
12-KLCA	0.0397 (0.0289, 0.0546)	0.0608 (0.0335, 0.1103)	0.0253 (0.0170, 0.0376)	0.0398 (0.0237, 0.0667)	0.0249 (0.0173, 0.0358)	0.0101 (0.0000, 0.0943)

The values in patients with liver disease are the least square geometric mean concentrations with 95% CI.

The values in healthy controls are the median with 2.5% point and 97.5% point.

^**^ *P* < 0.01 and ^*^ *P* < 0.05 versus viral hepatitis in patients with liver disease.

**(a):** 

(%)	Viral hepatitis (HBV + HCV)	ALD	Biliary tract disease	NAFLD	Other liver diseases
ALT	53.7	38.1	70.0	46.2	37.9
AST	52.2	61.9	55.0	23.1	44.8
ALP	43.3	57.1	70.0	30.8	55.2
Γ-GTP	34.3	81.0	90.0	15.4	51.7
T-Bil	46.3	47.6	80.0	61.5	48.3
D-Bil	44.8	55.0	78.9	50.0	33.3
TBA	57.6	52.4	52.6	16.7	42.9
ALB	55.2	42.9	20.0	84.6	42.9
PT	42.9	41.5	57.1	57.9	61.5

**(b):** 

(%)	Viral hepatitis (HBV + HCV)	ALD	Biliary tract disease	NAFLD	Other liver diseases
*n*	67	21	20	13	29

CA	53.7	61.9	35.0	46.2	44.8
GCA	44.8	71.4	60.0	23.1	51.7
TCA	46.3	61.9	65.0	23.1	51.7
CDCA	58.2	71.4	20.0	53.8	34.5
GCDCA	52.2	61.9	60.0	23.1	41.4
TCDCA	50.7	57.1	60.0	23.1	48.3
DCA	56.7	38.1	25.0	84.6	44.8
GDCA	53.7	42.9	60.0	53.8	37.9
TDCA	55.2	42.9	65.0	30.8	41.4
LCA	64.2	38.1	40.0	53.8	31.0
GLCA	62.7	28.6	35.0	53.8	44.8
TLCA	55.2	42.9	65.0	38.5	37.9
UDCA	61.2	66.7	25.0	23.1	41.4
GUDCA	61.2	61.9	25.0	23.1	44.8
TUDCA	59.7	57.1	25.0	23.1	51.7
12-KLCA	62.7	42.9	25.0	61.5	37.9
